# Body Composition Differentiates Prediabetes Phenotype Clusters in the Diabetes Prevention Program Study

**DOI:** 10.1210/clinem/dgaf163

**Published:** 2025-03-11

**Authors:** Benjamin M Stroebel, Meghana Gadgil, Kimberly A Lewis, Kayla D Longoria, Li Zhang, Elena Flowers

**Affiliations:** Department of Physiological Nursing, School of Nursing, University of California, San Francisco, CA 94143, USA; Department of Medicine, Division of General Internal Medicine, University of California, San Francisco, CA 94143, USA; Department of Physiological Nursing, School of Nursing, University of California, San Francisco, CA 94143, USA; Department of Physiological Nursing, School of Nursing, University of California, San Francisco, CA 94143, USA; Department of Medicine, Division of Hematology and Oncology, University of California, San Francisco, CA 94143, USA; Department of Epidemiology and Biostatistics, University of California, San Francisco, CA 94143, USA; Department of Physiological Nursing, School of Nursing, University of California, San Francisco, CA 94143, USA; Institute for Human Genetics, University of California, San Francisco, CA 94143, USA

**Keywords:** type 2 diabetes, clustering models, body composition, prediabetes

## Abstract

**Context:**

Type 2 diabetes (T2D) remains a significant public health problem, and current approaches to risk reduction fail to adequately prevent T2D in all individuals.

**Objective:**

The purpose of this study was to apply clustering methods that include metabolic risk factors and body composition measures to identify and characterize prediabetes phenotypes and their relationships with treatment arm and incident T2D.

**Design:**

Secondary analysis of the Diabetes Prevention Program clinical trial.

**Setting:**

Previously completed Diabetes Prevention Program trial.

**Patients or Other Participants:**

Subset of participants (n = 994) with body composition measures.

**Interventions:**

Not applicable.

**Main Outcome Measures:**

Unsupervised k-means clustering analysis was applied to derive the optimal number of clusters of participants based on common clinical risk factors alone or common risk factors plus more comprehensive measures of glucose tolerance and body composition.

**Results:**

Five clusters were derived from both the common clinical characteristics and the addition of comprehensive measures of glucose tolerance and body composition. Within each modeling approach, participants showed significantly different levels of individual risk factors. The clinical only model showed higher accuracy for time to T2D; however, the more comprehensive models further differentiated an overweight phenotype by overall metabolic health. For both models, the greatest differentiation in determining time to T2D was in the metformin arm of the trial.

**Conclusion:**

Data-driven clustering of patients with prediabetes allows for identification of prediabetes phenotypes at greater risk for disease progression and responses to risk reduction interventions. Further investigation into phenotypic differences in treatment response could enable better personalization of prediabetes and T2D prevention and treatment choices.

The global prevalence of type 2 diabetes (T2D), prediabetes, and related comorbidities continues to increase (1). Currently, the determination of prediabetes, which is a major risk factor for T2D, relies on the use of either hemoglobin A1c (HbA1c) or fasting blood glucose (FBG) (2), assigning patients a dichotomous classifier. However, significant mechanistic heterogeneity has been demonstrated in both T2D and prediabetes, with recent studies identifying subphenotypes that may confer different degrees of risk for T2D progression and adverse outcomes (3-10). This evidence suggests that the dichotomous classification approach is inadequate for detecting potential differences in the underlying etiology, progression to T2D, variability in disease severity and progression, and potential for responses to risk reduction interventions. Advancements in the characterization and understanding of the heterogeneity of T2D and prediabetes have the potential to increase precision in risk assessment and treatment.

Several prior studies identified subphenotypes of individuals with T2D based on clustering of clinical variables (3-8, 11), which may have implications for treatment, management, and prevention of diabetes-related complications. However, few prior studies focused on cluster phenotypes in the prediabetes window, during which time intensive risk reduction interventions have the potential to prevent T2D and related complications. In cohorts from the United Kingdom and Sweden, individuals with prediabetes were clustered according to 2-hour plasma glucose, insulin secretion, fasting insulin, waist circumference, hip circumference, body mass index (BMI), high-density lipoprotein cholesterol (HDL-C), and triglycerides. These studies resulted in the identification of 6 phenotypes among individuals with prediabetes, with differing risk for development of T2D and chronic kidney disease (6). This approach was repeated in a more diverse cohort from the National Health and Nutrition Examination Survey study. Individuals with prediabetes were clustered according to age at prediabetes diagnosis, BMI, HbA1c, FBG, 2-hour plasma glucose, homeostatic model assessment-insulin resistance (HOMA-IR), and homeostasis model assessment of β-cell function (HOMA-B), triglycerides, HDL-C, aspartate transaminase (AST), alanine transaminase (ALT), and glutamyl-transpeptidase, resulting in 6 clusters with different cardiac and renal outcome risks (7).

While all of the prior studies included common measures of body composition, none included more granular estimates of adipose tissue depots. Visceral adipose tissue is known to be metabolically more harmful than subcutaneous adipose tissue (12, 13), but differentiation between the 2 is not readily measured by BMI or waist circumference (14). The addition of measures of ectopic fat depot mass has the potential to further differentiate subphenotypes of individuals with prediabetes and associated risk for progression to T2D. There is the possibility for a proxy measure for the underlying body composition risk profile that can be identified using specific combinations of common risk factors based on underlying statistical associations with these more granular body composition measures.

Prior studies have also not evaluated whether subphenotypes are related to responses to risk reduction interventions. It is increasingly recognized that there are differences in individual responses to interventions that result from both biological (eg, genetic) and social-behavioral (eg, food insecurity) characteristics. These more granular subphenotypes have the potential to elucidate not only risk profiles that result from individual differences but also the likelihood of response to a given risk reduction intervention.

The Diabetes Prevention Program (DPP) was a landmark clinical trial that compared an intensive lifestyle intervention and metformin to placebo for the prevention of incident T2D (15). Building on emerging evidence about subphenotypes, the present study applies clustering methods to identify and characterize prediabetes phenotypes that include measures of ectopic fat. We also evaluated the relationships between these clusters and treatment arm and incident T2D in the DPP trial.

## Research Design and Methods

### Participants and Study Design

This study was a secondary analysis of data from participants in the DPP trial, which has been described in detail (16-18). Briefly, participants were recruited from 27 centers across the United States between 1996 and 1999, with oversampling from racially minoritized groups (18). Inclusion criteria required participants to be >25 years of age and have a BMI >24 kg/m^2^, a FBG 95 to 125 mg/dL, and a 2-hour postchallenge glucose 140 to 199 mg/dL. Exclusion criteria included use of medications known to alter glucose tolerance or serious illness. A total of 3234 participants were enrolled. The study described in this manuscript includes a subset of participants (n = 994) who underwent abdominal computerized tomography imaging and had measures of body composition available. We included data collected at baseline and diabetes outcome data collected throughout the DPP trial through the primary endpoint at 2 years.

### Demographic and Clinical Data Collection

DPP participant demographic characteristics, medical history, and FBG were collected by trained study personnel at the first screening visit (18). Sex and gender were reported as sex in the DPP trial, and therefore we have used that term in this paper as well. Physical measurements, oral glucose tolerance tests, behavioral data, and other laboratory tests were collected at the second interview screening visit (18). History and physical exams were collected at the third screening visit (18). Blood was collected by venipuncture according to a standardized protocol.

### Statistical Analyses

All statistical analyses were performed using R (version 4.3.2) (19). Data were summarized using descriptive statistics (means/medians and SDs/interquartile ranges for continuous variables and counts and percentages for categorical variables). HOMA-IR was calculated using the formula (fasting insulin × fasting glucose)/405, and HOMA-B was calculated using the formula 20 × fasting insulin/(fasting glucose −63). A ratio variable was created for AST/ALT. To compare between clusters, we used chi-squared (categorical variables) or ANOVA (continuous variables) to evaluate the demographic and clinical characteristics of the participants. Box and whisker plots were created to visualize the relative differences in clinical variables by cluster.

Clustering model variables were selected based on a priori knowledge about T2D risk factors, variables included in other clustering analysis studies that were also available in the DPP sample, and mechanistic relationships to metabolism and insulin signaling (3-8). We developed 2 models for clustering of participants. The clinical model included routinely measured clinical characteristics and risk factors (ie, age, BMI, FBG, HbA1c, HDL-C, triglycerides, and waist circumference). The clinicalPLUS + model included all the aforesaid variables, with the addition of measures for glucose tolerance and body composition (ie, 2-hour glucose, ALT, AST, HOMA-B, HOMA-IR, L2-L3 subcutaneous fat area, and L2-L3 visceral fat area) given their associations with mechanisms involved in body composition, metabolism, and insulin signaling (3-8). All variables used in clustering models were collected at trial enrollment and were centered to a mean value of 0 and a SD of 1.

Unsupervised k-means clustering was conducted for both the clinical and clinicalPLUS + variables for a range of cluster counts (k = 1-10). The average silhouette width, which ranges from −1 to 1 (1 indicating well-matched clusters), was calculated for each K value using the factoextra package in R to assess cluster stability and similarity within each cluster (20). The silhouette widths supported K values of 2, 4, and 5 (average silhouette widths 0.19, 0.18, and 0.16) in the clinical model and 2, 3, and 5 (average silhouette widths 0.17, 0.13, 0.12) in the clinicalPLUS + model as best fitting the data. We selected cluster counts of 5 for both the clinical and clinicalPLUS + models, as this choice provided a clearer delineation between cluster profiles across the clustering characteristics. We also performed a stratified analysis by sex for the clinical model using the same methods.

Five-cluster k-means models were subsequently created for both the clinical and clinicalPLUS + models using the Cluster package in R (21). Cluster labels were assigned based on the mean values of each variable included in the clusters.

Kaplan–Meier (KM) survival estimates and overall log-rank tests with post hoc tests adjusted for multiple comparisons via Bonferroni–Holm corrections were used to assess differences in time to T2D development between clusters in the clinical and clinicalPLUS + models, both overall and by DPP treatment arm. Cox proportional hazard (CPH) models adjusted for DPP treatment, participant sex, race, and ethnicity were used to determine hazard ratios (HRs) for time to T2D at the primary trial endpoint of 2 years by cluster. K-fold cross-validation was used to compute c-index scores for the fully adjusted clinical and clinicalPLUS + Cox models using the glmnet package for R (22, 23).

## Results

### Cluster Characteristics and Labels

**Figure 1.**
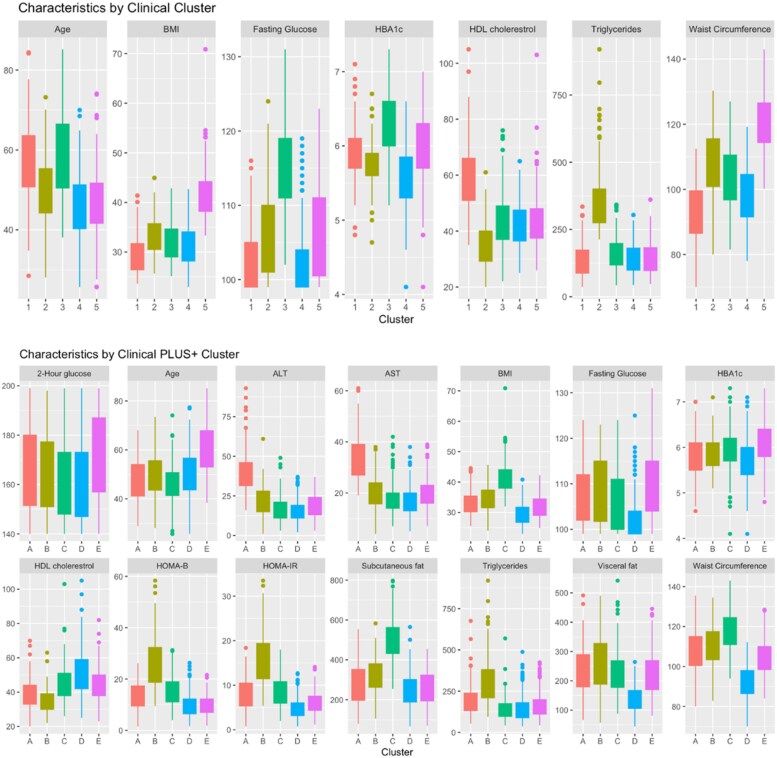
Distribution of clinical risk factors included in phenotype models. Box and whisker plots: each plot shows the distribution of a clinical risk factor by phenotype cluster. Within each box, the bottom border represents the 25th percentile and the upper border represents the 75th percentile. The lowest point on the vertical line represents the minimum and the highest point represents the maximum. Small circles represent outliers. Phenotype groups for the clinical model: 1, older; 2, dyslipidemia; 3, insulin resistant; 4, younger protected; 5, higher adiposity. Phenotype groups for the clinicalPLUS + model: A, hepatic steatosis; B, dyslipidemia-insulin resistance, C, subcutaneous adipose; D, protected; E, older dysglycemia. The y-axis shows units as described: 2-hour glucose, mg/dL; age, years; ALT, IU/L; AST, U/L; BMI, kg/m^2^; fasting glucose, mg/dL; HbA1c, percent; HDL cholesterol, mg/dL; HOMA-B; HOMA-IR; subcutaneous fat, cm^2^/m^2^; triglycerides, mg/dL; visceral fat, cm^2^/m^2^; waist circumference, cm. Abbreviations: ALT, alanine transaminase; AST, aspartate aminotransferase; BMI, body mass index; HbA1c, hemoglobin A1c; HDL, high-density lipoprotein; HOMA-B, homeostasis model assessment of β-cell function; HOMA-IR, homeostasis model assessment of insulin resistance.

Five distinct clusters were identified and labeled in the clinical cluster models: (1) older, with the highest proportion of females; highest HDL-C and second highest age; and lowest BMI, triglycerides, and FBG; (2) dyslipidemia, the smallest cluster with the lowest proportion of females, characterized by the highest triglycerides and lowest HDL-C; (3) insulin resistant, with the highest age, FBG, and HbA1c; (4) younger protected, the largest cluster, featuring the lowest age, FBG, and HbA1c and relatively lower BMI and waist circumference; and (5) higher adiposity, with the second highest proportion of females, highest BMI and waist circumference, and relatively lower triglycerides (Fig. 1). All clinical clusters differed significantly in their sex and race and ethnicity composition [Supplementary Table S1 (24)]. In a stratified analysis by sex, we found that clusters for women and men had generally similar characteristics [Supplementary Table S4 (24)] with very little cross-over between sex-stratified clusters compared to the sample overall [Supplementary Table S5 (24)]. Cox proportional hazards models also showed that risk for incident T2D was similar within cluster profiles for both men and women compared to the sample overall. The main difference was in males in which the dyslipidemia group was further differentiated in the high triglycerides (442 mg/dL) and low HDL (30 mg/dL) vs relatively lower risk profile (163 mg/dL, 38 mg/dL) with similar values for other characteristics [Supplementary Table S4 (24)].

Five distinct clusters were also identified and labeled in the clinicalPLUS + clustering models (labeled with letters rather than numbers to differentiate them from the clinical clusters): (A) hepatic steatosis, the lowest proportion of females, lower HDL-C, higher triglycerides, high visceral fat area, and highest AST and ALT; (B) dyslipidemia-insulin resistance, the smallest cluster with the lowest HDL-C and highest triglycerides, visceral fat area, HOMA-IR, and HOMA-B; (C) subcutaneous adipose, a predominantly female cluster with the lowest age, highest BMI and subcutaneous fat area, lowest triglycerides and 2-hour glucose, with higher HOMA measures and HbA1c; (D) protected, the largest cluster with the highest proportion of women and the lowest BMI, waist circumference, visceral and subcutaneous fat areas, FBG, AST, ALT, HbA1c, and HOMA-measures and highest HDL-C; and (E) older dysglycemia, the oldest cluster featuring lower BMI, waist circumference, HOMA-IR, and subcutaneous fat area and the highest FBG, 2-hour glucose, HbA1c, and HOMA-B (Fig. 1). All clinicalPLUS + clusters differed significantly in their sex and race and ethnicity composition [Supplementary Table S1 (24)]. The comparisons in cluster membership between clinical and clinicalPLUS + are shown in Supplementary Table S2 (24). In a stratified analysis by sex, we found moderate cross-over between sex-stratified clusters compared to the sample overall [Supplementary Table S5 (24)]. Only for males was there a hepatic steatosis group (male A) whereas females had generally healthier liver function. Both women and men had an older dysglycemia cluster (female C, male C) and dyslipidemic clusters (female C, male A, male B) One cluster of females (female E) was more obese with highly elevated subcutaneous fat but relatively lower visceral fat area, whereas for men, 1 cluster had high levels of both subcutaneous and visceral fat area (male D) [Supplementary Table S4 (24)].

### Time to Incident T2D—Clinical Clusters

**Figure 2.**
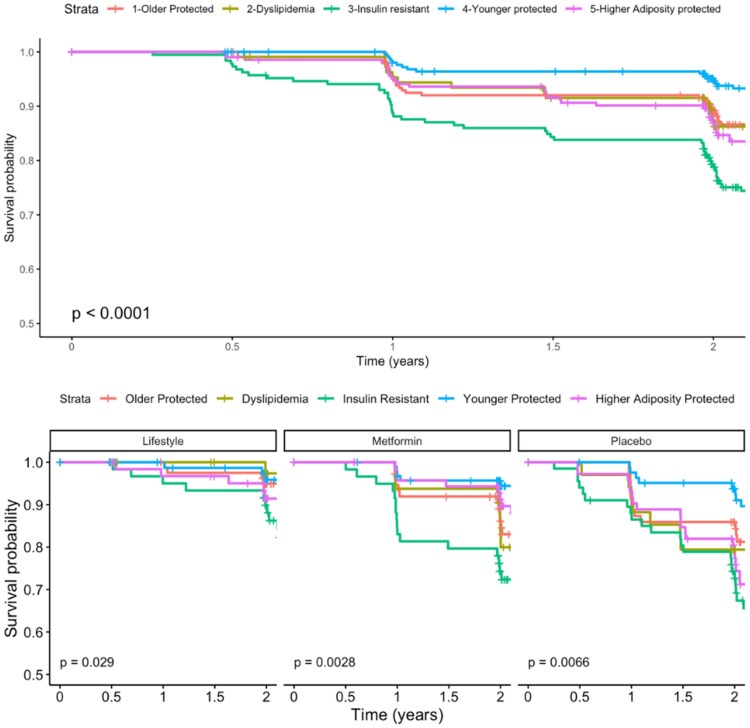
Survival curves for incident T2D in the clinical cluster model. Curves represent the proportion of the sample free from T2D at a given time point. Data were censored at the primary trial endpoint of 2 years. Abbreviation: T2D, type 2 diabetes.

Time to T2D differed significantly between clinical clusters, both overall and by DPP treatment arm, in univariate KM analyses (Fig. 2, *P* < .05). Post hoc tests indicated that time to T2D development was significantly lower overall in several comparisons: insulin resistant (cluster 3) vs older (cluster 1), older (cluster 1) vs younger protected (cluster 4), insulin resistant (cluster 3) vs dyslipidemia (cluster 2), dyslipidemia (cluster 2) vs younger protected (cluster 4), insulin resistant (cluster 3) vs younger protected (cluster 4), insulin resistant (cluster 3) vs higher adiposity (cluster 5), and higher adiposity (cluster 5) vs younger protected (cluster 4) (adjusted *P* < .05). Time to T2D was also significantly lower in the placebo arm in the insulin-resistant (cluster 3) vs younger protected (cluster 4) and in higher adiposity (cluster 5) vs younger protected (cluster 4) (adjusted *P* < .05). In the metformin arm, time to T2D was significantly lower in the insulin-resistant (cluster 3) vs younger protected (cluster 4) (adjusted *P* < .05). Time to T2D did not differ significantly between phenotype clusters in the lifestyle arm in post hoc tests (adjusted *P* > .05). Adjusted Cox models showed higher HR (95% confidence interval) for T2D, relative to younger protected (cluster 4), in older (cluster 1) [2.22 (1.20, 4.11)], dyslipidemia (cluster 2) [2.32 (1.13, 4.77)], insulin resistant (cluster 3), [4.93 (2.77, 8.75)], and higher adiposity (cluster 5) [2.76 (1.51, 5.03)] (Table 1). The fully adjusted CPH model with clinical clusters achieved a Brier score of 0.100 and a c-index score of 0.68, and the proportional hazards assumption was met. In terms of overall prevalence, the highest risk groups had 3-fold higher estimates compared to the lowest risk groups (eg, 26% in the insulin-resistant (3) group compared to 6% in the youngest and lowest risk group) [Supplementary Table S3 (24)]. In the sex-stratified analysis, concordant cluster risk profiles were associated with risk for T2D compared to the overall sample, including by trial arm [Supplementary Table S6 (24)].

**Table 1.** Hazards ratios for incident T2D by cluster

Cluster	HR	95% CI	*P*-value
Clinical model			
Older	2.22	1.20, 4.11	.0113
Dyslipidemia	2.32	1.13, 4.77	.0215
Insulin resistant	4.93	2.77, 8.75	<.0001
Younger protected	Ref	NA	NA
Higher adiposity	2.76	1.51, 5.03	.001
Clinical PLUS + model			
Hepatic steatosis	2.01	1.07, 3.77	.0302
Dyslipidemia-insulin resistance	1.92	0.96, 3.86	.0644
Subcutaneous-adipose	1.77	1.07, 2.92	.0258
Protected	Ref	NA	NA
Older dysglycemia	3.03	1.89, 4.86	<.0001

Abbreviations: CI, confidence interval; HR, hazards ratio; NA, not available.

### Time to Incident T2D—ClinicalPLUS + Clusters

**Figure 3.**
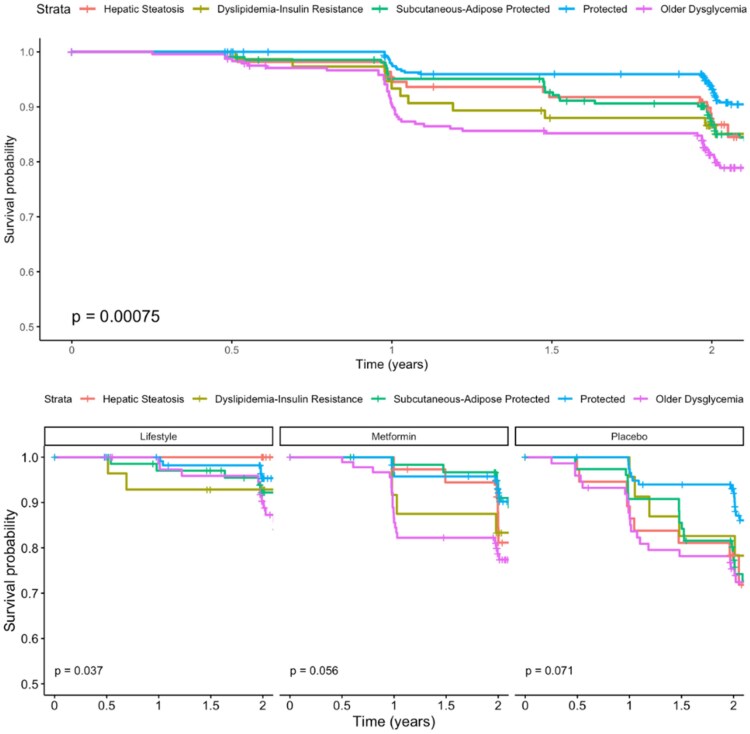
Survival curves for incident T2D in the clinicalPLUS + cluster model. Curves represent the proportion of the sample free from T2D at a given time point. Data were censored at the primary trial endpoint of 2 years. Abbreviation: T2D, type 2 diabetes.

Time to T2D differed significantly between clinicalPLUS + clusters overall and in the lifestyle treatment arm (Fig. 3, *P* < .05). Post hoc tests indicated that time to T2D development was significantly lower in older dysglycemia (cluster E) compared to protected (cluster D) (adjusted *P* < .05). Adjusted Cox models indicate that the HR (95% confidence interval) for T2D, relative to protected (cluster D), was 2.01 (1.07, 3.77) for hepatic steatosis (cluster A), 1.92 (0.96, 3.86) for dyslipidemia-insulin resistance (cluster B), 1.77 (1.07, 2.92) for subcutaneous adipose (cluster C), and 3.03 (1.89, 4.86) for older dysglycemia (cluster E) (Table 1). The fully adjusted CPH model with clinicalPLUS + clusters achieved a Brier score of 0.123 and a c-index of 0.61, and the proportional hazards assumption was met. As with the clinical models, an evaluation of total prevalence after 2 years showed drastically higher estimates in the higher risk groups [eg, 21% in the older dysglycemia (E) cluster compared to 9% in the protected (D) cluster] [Supplementary Table S3 (24)]. The clinical and clinicalPLUS + models differentiated risk for incident T2D by clusters with similar accuracy [Supplementary Table S6 (24)]. KM curves showed greater differentiation for incident T2D in the sex-stratified models across all 5 clusters for the placebo group, and, unlike in the sample overall, there were 2 groups (female B, female D) that were more responsive to metformin in females, although this did not reach clinical significance [Supplementary Table S7 (24)]. For males, the most dysglycemic group (male C) had the highest incidence of T2D in the metformin group [Supplementary Table S7 (24)].

## Discussion

Using data from the DPP trial, we applied an unsupervised clustering algorithm and identified 2 sets of 5 clusters among participants with prediabetes based on 7 (clinical) and 14 (clinical PLUS+) phenotypic variables. Our study demonstrated distinct differences in phenotypic characteristics and rates of progression to T2D between clusters. Relative time to T2D progression also differed according to DPP treatment arm, suggesting the effects of treatment on time to T2D progression may differ according to baseline subphenotype. In addition, the overall prevalence of T2D varied significantly between clusters, with the highest risk groups exhibiting a 3-fold increase compared to the lowest risk groups [Supplementary Table S3 (24)]. Previously, based on a dichotomous prediabetes classification, these participants were considered to have similar risk profiles. Our findings highlight the potential value of identifying distinct subgroups to better capture the heterogeneity in prediabetes risk, which would allow for increased precision in risk assessment and treatment.

The clinical cluster model that relies on widely available variables demonstrated a slightly better Brier score compared to the clinicalPLUS + model. Consequently, the clinical cluster model may be more easily replicated in other datasets, highlighting its potential clinical utility as a risk stratification tool, especially in settings without access to advanced body composition measures.

As expected, the insulin resistant cluster 3 shows the greatest risk for incident T2D. The identified clinical dyslipidemia phenotype (cluster 2) shares a trend of high levels of triglycerides coupled with low HDL-C, also observed in patients with metabolic syndrome, has a moderate progression to T2D that validates this risk profile as being important even in the absence of obesity (25). Similarly, the higher adiposity phenotype (cluster 5), with the highest BMI and waist circumference and more moderate FBG and HbA1c, resembles the phenotype of patients with metabolically benign obesity and may inform treatment decisions regarding weight management, insulin sensitivity, and lipid status (26). The older cluster also shows a more moderate rate of progression to T2D, which is consistent with prior data that show that a diagnosis of prediabetes in older age (ie, > 65 years) has a lower rate of progression to T2D compared to a younger age at diagnosis (27). We also noted a higher proportion of women in the higher adiposity (cluster 5) and older (cluster 1) phenotypes.

Characterization of clinicalPLUS + cluster phenotypes revealed significant differences in underlying metabolic states that could inform further studies on the mechanistic underpinnings of prediabetes and progression to T2D. Phenotypic differences in transaminases, body composition, insulin resistance, and β-cell function have been previously described in phenotypic clusters of individuals with prediabetes (6, 7). Our study noted significant differences in the relative area of visceral fat and subcutaneous fat between clusters in the clinicalPLUS + clustering model. By traditional measures of body composition (ie, BMI, waist circumference), cluster C was the most overweight/obese. However, upon more granular differentiation of adipose tissue depots, the subcutaneous adipose phenotype (cluster C) exhibited elevated L2-L3 subcutaneous fat and a comparatively longer time to T2D relative to the dyslipidemia-insulin resistance phenotype (cluster B), which also showed higher BMI and waist circumference compared to other groups but had elevated L2-L3 visceral fat area (vs subcutaneous). These observed differences suggest a dynamic role for body composition, consistent with prior evidence that links visceral fat volume with that of pancreatic fat, which has previously been associated with altered insulin secretion in individuals with prediabetes (6). For the clinicalPLUS + models, we again noticed a marked difference in the proportion of women, particularly in the subcutaneous adipose (84%, cluster C) and protected (85%, cluster D) groups compared to the other groups but here with no distinct differences in mean age other than in the older dysglycemia group (44%, cluster E).

In both the clinical and clinicalPLUS + clusters, metformin was markedly more impactful in clusters that were characterized by cardiometabolic risk factors other than insulin resistance and dysglycemia. For the clinical models, the older (cluster 1), dyslipidemia (cluster 2), and higher adiposity (cluster 5) showed the greatest reduction in incident T2D in the metformin group compared to placebo, with relatively little improvement in the insulin-resistant group (cluster 3). For the clinicalPLUS + models, it is the hepatic steatosis (cluster A) and subcutaneous adipose (cluster C) that show the greatest reduction in incident T2D, with the dyslipidemia-insulin resistance (cluster B) and older glycemia (cluster E) clusters showing relatively little change. The mechanisms underlying the impact of metformin are not fully understood, and recent evidence suggests that this agent may have benefits beyond diabetes prevention (28, 29). The observation that metformin response varies by subphenotype suggests the potential for individualized treatment approaches to optimize risk reduction. In addition, there is a sharp increase in incidence of T2D after 1 year for clinical clusters 3 and clinical PLUS + clusters B and E in the metformin group, which may have clinical implications for risk stratification. In contrast, for both clustering approaches, all groups showed benefit from the intensive lifestyle intervention, which supports continued efforts to identify strategies for pragmatic implementation of lifestyle and behavior change interventions that may include policy- and social-level implications.

Similar to results in a previous clustering analysis of National Health and Nutrition Examination Survey participants with prediabetes (7), the distribution of the clusters identified in this study varied by race and ethnicity. Cluster 2 in the Jiang et al analysis had a higher proportion of individuals categorized as Black and the highest BMI, which is similar to clinical cluster 5 (higher adiposity) in our study. Evidence suggests that the common measures of body composition (eg, BMI, waist circumference) may not provide equal information about risk between race and ethnic groups (30). For example, clinicalPLUS + cluster C (subcutaneous adipose) had high BMI, waist circumference, and subcutaneous adipose tissue but relatively lower visceral adipose tissue and is somewhat protected from incident T2D compared to the hepatic steatosis cluster (cluster A) that had lower measures of BMI and waist circumference but higher visceral adipose tissue and a smaller proportion of people categorized as Black. Clusters 1 and 2 from the Jiang et al paper were the youngest with the highest proportions categorized as Mexican-American or other Hispanic, with cluster 1 showing relatively healthier insulin and glucose metabolism, whereas cluster 2 had some of the poorest measures of insulin and glucose metabolism. In our study, the younger protected cluster (cluster 4) had the highest proportion of people categorized as Hispanic, similar to Jiang et al's cluster 1. The second highest proportions were in the dyslipidemia (cluster 2) and insulin-resistant (cluster 3) clusters, which may overlap with Jiang et al's cluster 2 and suggest that there may be important differences in risk for T2D based on subphenotypes within race and ethnic group categorization. These observations highlight important potential contributions to risk for T2D that may result from both genetic differences determined by geographical ancestry as well as social implications of race and ethnic group categorization.

While the overall cluster characteristics for the clinical clusters remained consistent in a stratified analysis by sex, we found differences in the overall clusters in the proportion of women, many of which support what is known about sex differences related to these risk factors. In general, women develop cardiometabolic risk later in life and have a higher proportion of less harmful subcutaneous adipose tissue and higher HDL-C compared to men, which matches what is observed in the older (cluster 1) and higher adiposity (cluster 5) clinical models and has moderate hazards for T2D compared to the highest and lowest risk clusters. The same pattern was evidenced in the clinicalPLUS + model where subcutaneous-adipose cluster C that included 84% women had markedly higher levels of subcutaneous adipose tissue compared to any of the other clusters and yet showed moderate overall or even the lowest hazards for incident T2D in the metformin arm. These findings are in contrast to the Jiang et al paper in which cluster 6 had the highest proportion of women but the lowest, almost normal BMI. These findings further support the need for careful classification of risk factors in order to optimize risk stratification, and particularly for women, in which basic clinical measures of body composition may inflate true risk for T2D compared to other risk profiles.

### Limitations

Selection of variables to include in the clustering models reflected previous studies, but an optimal set of clustering characteristics and the ideal clustering models have yet to be established. Current clustering methods primarily upon phenotypic variables, which may be confounded, mediated, or otherwise influenced by socioenvironmental factors related to T2D progression. Cluster assignment is likely dynamic rather than static, highlighting the need for further studies to understand how phenotypes may evolve over time or change in response to risk reduction interventions and socioenvironmental factors. Comparisons between men and women are limited by how the data were collected and reported in the DPP trial and may impact whether biological sex is concordant with the sex variable group assignment.

As efforts to individualize treatment and management of prediabetes continue, working toward more precise stratification of risk for progression to T2D becomes increasingly important. Data-driven clustering of patients with prediabetes works toward this end, allowing for identification of subphenotypes at greatest risk for disease progression and responses to risk reduction interventions. This secondary analysis of participants from the DPP trial who had prediabetes applied a data-driven approach to identify 2 models to cluster participants based on phenotypic characteristics. The clinical clusters leverage commonly measured risk factors for T2D and differentiated between subgroups based on age, insulin metabolism and dyslipidemia profiles, and anthropometric measures of body composition. In general, the youngest group with the healthiest risk factor profile had significantly lower rates of T2D, while the most insulin-resistant group had the highest, regardless of trial arm. The addition of more refined estimates of glucose and insulin metabolism and adipose tissue composition confirmed prior studies that showed type of adipose tissue, and not just BMI, differentiates risk for T2D. Overall, the intensive lifestyle intervention was effective at decreasing risk for T2D regardless of phenotype cluster, whereas some clusters showed markedly better response to metformin compared to others. We also observed differences that suggest further research is needed to determine how identified subphenotypes of prediabetes are related to ancestry vs race and ethnic group categorization and between biological sexes and risk for T2D. Further investigation into phenotypic differences in treatment response could enable better personalization of prediabetes and T2D prevention and treatment choices. This is especially salient given the addition of newer therapeutics like GLP-1 receptor agonists where cost may factor into patient acceptability when compared to older therapeutics, which may still offer phenotype-specific efficacy (31).

## Data Availability

All datasets analyzed during the current study are publicly available through the NIDDK data repository.
